# Obstetric Case, Complicated with an Encysted Tumor of the Uterus

**Published:** 1844-07-13

**Authors:** James Anderson


### RECORD OF MEDICAL SCIENCE.

### OBSTETRIC CASE, COMPLICATED WITH AN ENCYSTED TUMOR OF THE UTERUS.

BY JAMES ANDERSON, ESQ.

On the 16th September, 1831, Andrew Ferguson, M. D., Aberdeen, called on me to accompany him to see a patient of his, a Mrs. S——n, Netherkirkgate, (pregnant, if I am not mistaken, with her third child); the Dr. and a mid-wife had been in attendance upwards of two days; the parturient pains had been regular and strong; nevertheless the os uteri was little dilated and rigid; the pains on my visit suspended. Feverish—pulse 98. On passing the hand over the abdomen, two distinct tumours were felt, one superior, and the other inferior, divided transversely by a deep contraction, as with a circular band, at the umbilicus; the inferior feels elastic; no motion in the other; the fœtus in utero was distinctly recognised through the abdominal parietes. I abstracted blood from the arm, emptied the bladder, exhibited an enema, and administered some cordial medicine; applied a roller moderately tight below the epigastric region; the pains soon returned; the os uteri gradually lost its rigidity, and dilated, so that I could introduce my finger, and assist in the dilatation; the inferior tumour began to descend, which we conceived to be the amnios; however it proved not to be the case, for as soon as I gave over the dilatation and examination, a discharge immediately followed, of purulent matter, to the amount of about seven pounds' weight; the bed-room became so impregnated with noxious gas, that neither the patient nor attendants could have withstood it, if immediate means had not been resorted to for its purification; the liquor amnii discharged, and I delivered her of a fine healthy female child, at full time; there was little uterine hæmorrhage; the placenta came away without much trouble, bringing along with it the cyst of the tumour—*this was an encysted tumour within the uterus*; no disease could be detected in the uterine cavity—no leucorrhœal discharge, &c.; no pain in coitu, notwithstanding purulent matter is indicative of previous inflammation; yet, during the whole period of gestation, she was in her usual state of health, and had a good recovery.—*London Medical Times.*

